# Unraveling the Lipidomic Determinants of Atrial Fibrillation: An Extensive Mendelian Randomization Study

**DOI:** 10.2174/0118715303378914250418095928

**Published:** 2025-04-29

**Authors:** Lingyan Ye, Dongli Lin, Feng Chen, Xiaoyong Huang, Xianjun Wu

**Affiliations:** 1 Department of Cardiology, Lishui People's Hospital, the Sixth Affiliated Hospital of Wenzhou Medical University, Lishui, Zhejiang, China;; 2 Department of Cardiology, First Affiliated Hospital of Lishui University School of Medicine, Lishui, Zhejiang, China

**Keywords:** Mendelian randomization, atrial fibrillation, lipidome, genetic epidemiology, cardiovascular genetics, causal inference

## Abstract

**Background:**

Atrial Fibrillation (AF) is the most prevalent form of cardiac arrhythmia, with a complex etiology that implicates lipid metabolism. This study employs Mendelian Randomization (MR) to dissect the causal relationships between lipidomic profiles and AF, utilizing comprehensive genetic data to clarify these associations.

**Methods:**

Summary statistics for 179 lipid species across 13 classes were retrieved from the GWAS Catalog, encompassing 7,174 Finnish individuals from the GeneRISK study. For AF, data were synthesized from six major studies comprising over one million subjects. Our Two-Sample MR (TSMR) approach was implemented using Inverse Variance Weighting (IVW), MR-Egger, and MR-PRESSO for sensitivity analysis. Additionally, we uniquely integrated the Mendelian Randomization-Bayesian Model Averaging (MR_BMA) method to robustly prioritize the most likely causal lipid determinants of AF, and performed bidirectional MR analysis to assess potential reverse causality.

**Results:**

The TSMR analysis, reinforced by MR_BMA, revealed significant causal associations between specific lipid species and AF risk. In particular, Phosphatidylcholine (17:0_18:2) was associated with a decreased risk of AF (OR = 0.96, 95% CI 0.93–0.99, *P*<0.05), whereas Phosphatidylcholine (16:0_20:5) and Phosphatidylcholine (17:0_20:4) were linked to increased risks (OR = 1.04, 95% CI 1.01–1.07, *P*<0.01; and OR = 1.02, 95% CI 1.00–1.05, *P*<0.05, respectively). Furthermore, elevated levels of Phosphatidylethanolamine (18:0_20:4) (OR = 1.03, 95% CI 1.01–1.06, *P*<0.01) and Triacylglycerol (50:4) (OR = 1.04, 95% CI 1.00–1.07, *P*<0.05) were also associated with increased AF risk. In addition, Sphingomyelin (d34:2), Sterol ester (27:1/18:0), and Sterol ester (27:1/18:3) emerged as further risk factors, thereby expanding the spectrum of lipidomic determinants implicated in AF. The bidirectional MR analysis provided no evidence of reverse causation, reinforcing the directionality of the lipid-driven association. Sensitivity analyses demonstrated robust findings with no indication of pleiotropy or heterogeneity.

**Conclusion:**

This study provides strong evidence for thecausal role of specific lipid species in the development of AF. Our comprehensive MR analysis not only deepens our understanding of AF pathophysiology but also highlights the therapeutic potential of targeting these lipid alterations. Notably, the absence of reverse causation supports a unidirectional relationship wherein altered lipid species drive AF risk.

## 1 INTRODUCTION

Atrial Fibrillation (AF) remains a formidable challenge in cardiovascular medicine, affecting an estimated 59.70 million individuals worldwide [1]. The increasing prevalence of AF is a major concern due to its association with significant morbidity and mortality, particularly from stroke, heart failure, and systemic embolism [2]. Understanding the etiology of AF is critical, and recent insights suggest that genetic predispositions play a significant role in its pathogenesis [3].

The emergence of lipidomic profiling has revolutionized our understanding of the molecular underpinnings of AF. Lipidomics, which involves the comprehensive analysis of lipids within a biological system, has highlighted the crucial role of lipid metabolism in cardiovascular diseases [4, 5]. Lipid dysregulation has been implicated in the development of AF, positioning lipidomic alterations as potential biomarkers and therapeutic targets for this arrhythmia [6, 7].

Mendelian Randomization (MR) utilizes genetic variants as instrumental variables (IVs) to infer causal relationships between exposures and clinical outcomes, thereby overcoming confounding factors endemic in observational studies by leveraging the random allocation of alleles at conception [8]. Integrating MR with lipidomic analyses has enabled researchers to begin unraveling the causal pathways that link lipid metabolism to AF, thus enhancing our understanding of its mechanisms [9, 10].

However, comprehensive studies systematically evaluating the causal impact of lipidomic profiles on AF remain scarce. This gap hinders the development of effective prevention and treatment strategies informed by lipidomic evidence [11]. Our study aims to bridge this gap by employing a Two-Sample Mendelian Randomization (TSMR) approach [12], complemented by Bayesian Model Averaging Mendelian Randomization (MR_BMA) to robustly prioritize the most likely causal lipid determinants of AF [13, 14].

Incorporating bidirectional MR analysis, our study analyzedlarge-scale Genome-Wide Association Study (GWAS) datasets to explore the causal effects of various lipid species on AF. This bidirectional approach allows us to investigate not only the impact of lipid levels on AF but also the potential influence of AF on lipid species, providing a comprehensive view of their interplay. We assessedheterogeneity and horizontal pleiotropy through rigorous statistical methods and performedsensitivity analyses to ensure the robustness of our findings. By elucidating the lipid species landscape of AF, our research endeavors to make a significant contribution to the field of cardiovascular genetics and to open new avenues for novel therapeutic interventions.

## 2 METHODS

The research utilized a bidirectional approach to MR analysis. This method necessitated adherence to three foundational assumptions for SingleNucleotide Polymorphisms (SNPs), as depicted in Fig. (**1**).

### 2.1 Collection of LipidomeGWAS Summary Statistics

Summary statistics for lipidome traits were retrieved from the GWAS Catalog, with identifiers ranging from GCST90277238 to GCST90277416. The dataset encompassed 7,174 Finnish individuals from the GeneRISK study, all unrelated, to investigate the genetic underpinnings of 179 lipid species. These species are categorized into 13 lipid classes and fall under four main categories, as delineated in Supplementary Table **1** [15]. The 179 lipid species were selected both because they are comprehensively profiled in the GeneRISK GWAS dataset and due to their documented involvement in cardiovascular metabolism, as supported by previous studies [15-18].

### 2.2 Synthesis ofAtrial Fibrillation GWAS Meta-Analysis Data

Data on AF wereaggregated from a comprehensive GWAS meta-analysis spanning six prominent studies: The Nord-Trøndelag Health Study (HUNT), deCODE, the Michigan Genomics Initiative (MGI), DiscovEHR, UK Biobank, and the AFGen Consortium. This extensive analysis incorporated data from 60,620 AF cases alongside 970,216 control participants, all of European descent, with the dataset being accessed on April 28, 2021 [19].

### 2.3 Refinement of Instrumental Variables for Lipidome Impact on Atrial Fibrillation

In the pursuit of assessing the lipidomic influence on AF, we meticulously selected SNPs as IVs adhering to the foundational principles of MR. The IVs were chosen based on their robust genetic association with lipidomic profiles, evidenced by a significance threshold of *P* < 1×10^−5^, and their independence from confounding factors that could potentially distort the lipidome-AF nexus. To mitigate the confounding effects of Linkage Disequilibrium (LD), SNPs demonstrating an LD r^2^ value at or above 0.001 were systematically excluded. This was complemented by setting a genetic distance parameter of 10,000 kb to further eliminate SNPs within this range that surpassed the LD threshold, thereby ensuring the purity of the IVs set.

The PhenoScanner database was instrumental in the identification process, allowing for the exclusion of any SNPs havingdirect associations with AF, thus preserving the integrity of the MR analysis. The MR Pleiotropy RESidual Sum and Outlier (MR-PRESSO) test was then employed to scrutinize the selected SNPs for potential pleiotropic effects [20]. This rigorous evaluation confirmed the suitability of the SNPs, as none displayed indications warranting exclusion.

### 2.4 Enhanced TSMR Analysis with Reverse Directionality

The Inverse Variance Weighting (IVW) method stands as the cornerstone of our TSMR analysis, adeptly combining multiple random variables to minimize overall variance. This technique judiciously assigns weights based on the degree of variance among the variables, facilitating the synthesis of findings from disparate studies. Employing IVW as the primary analytical tool ensures the derivation of the most precise and reliable estimates, contingent upon the validity of all genetic variants as instruments [21]. To bolster the veracity of our results, the study incorporated a suite of methods, including MR-Egger and weighted median analyses. These methodologies act as bulwarks, providing robust and reliable outcomes by serving as additional safeguards [22]. The congruence of causal direction across multiple methods fortifies the confidence in the established causal relationship [23, 24]. Moreover, the study integrated reverse MR methods to account for potential reverse causality, addressing scenarios where the outcome might influence the exposure [25].

Additionally, the MR_BMA approach was harnessed to further substantiate the causal effects of the exposures on the outcome by reducing biases and concurrently modeling multiple correlated risk factors-a technique particularly advantageous for high-throughput datasets [14].

### 2.5 Rigorous Sensitivity Analysis for MR Findings

A meticulous sensitivity analysis was performed to affirm the robustness of the conclusion of our study.The initial step involved evaluating the MR-Egger regression model’s intercept to detect any potential pleiotropic effects [26]. An intercept term *P*-value greater than 0.05 would indicate a minimal influence of genetic pleiotropy, thereby supporting the premise that the IVs affect AF risk exclusively through lipidomic pathways. To delve into the heterogeneity of IVsand theirpossible impact on the causal estimate, the study utilized Cochran’s Q test [27]. This test is instrumental in identifying variability among the IVs that could potentially alter the causal inference.

Additionally, a leave-one-out analysis was conducted to further assess the sensitivity of the results. This process entailed performing the MR analysis iteratively, each time excluding a different SNP, to ensure the stability and reliability of the findings [28]. The MR-PRESSO test was subsequently applied to discern any discrepancies in the MR analysis outcomes, both before and after correction. The combined use of the MR-Egger intercept test and MR-PRESSO facilitated a multifaceted sensitivity analysis. While the MR-Egger intercept test detects the presence of directional pleiotropy, MR-PRESSO further identifies and corrects for outliers that may be contributing to such bias [20].

## 3 RESULTS

### 3.1 Causal Associations Between Lipidomic Profiles and Atrial Fibrillation

Our TSMR analysis revealed that various lipid categories-including glycerophospholipids, sphingolipids, glycerolipids, and sterols-exhibit significant associations with AF. Specifically, within the glycerophospholipids, certain phosphatidylcholines were linked to altered AF incidence, with their molecular structures influencing the direction of risk. For instance, Phosphatidylcholine (17:0_18:2) was associated with a decreased risk (OR = 0.96, 95% CI 0.93–0.99, *P*<0.05), while Phosphatidylcholine (16:0_20:5) and Phosphatidylcholine (17:0_20:4) showed increased risks (OR = 1.04, 95% CI 1.01–1.07, *P*<0.01; and OR = 1.02, 95% CI 1.00–1.05, *P*<0.05, respectively). Additionally, Phosphatidylethanolamine (18:0_20:4) emerged as a risk factor (OR = 1.03, 95% CI 1.01–1.06, *P*<0.01).

In the sphingolipid category, elevated levels of Sphingomyelin (d34:2) were indicative of increased AF development (OR = 1.05, 95% CI 1.02–1.08, *P*<0.001). Similarly, the glycerolipid Triacylglycerol (50:4) demonstrated a risk effect (OR = 1.04, 95% CI 1.00–1.07, *P*<0.05). Notably, sterol esters, such as Sterol ester (27:1/18:0) and Sterol ester (27:1/18:3), consistently indicated a heightened risk for AF (OR = 1.04, 95% CI 1.00–1.07, *P*<0.05; and OR = 1.05, 95% CI 1.02–1.09, *P*<0.01, respectively).

**Fig. (2):**
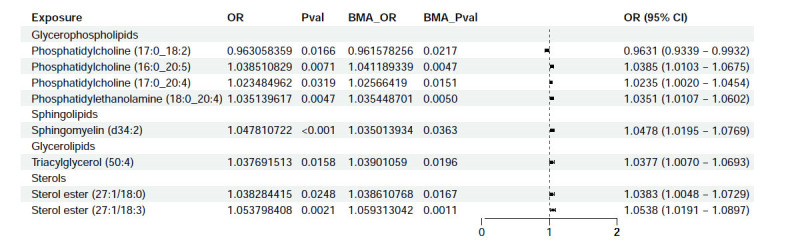
Forest plot comparing the causal effect estimates of lipidomic profiles on atrial fibrillation obtained using traditional Mendelian randomization *via* the IVW method and the Bayesian Model Averaging MR (MR_BMA) approach. The plot displays odds ratios (OR), *P*-values, and 95% confidence intervals (CI) derived from the IVW analysis, while the MR_BMA results (BMA_OR and BMA_Pval) further support the prioritization of causal lipid determinants. These estimates collectively underscore the robustness of the causal associations observed in our study.

**Fig. (3):**
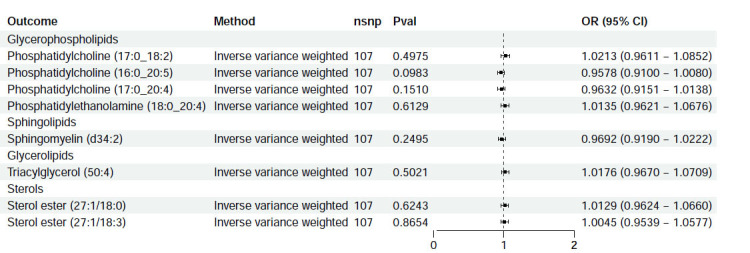
Forest plot of the reverse Mendelian randomization analysis assessing the effect of atrial fibrillation on lipidomic profiles. This analysis demonstrates that there is no evidence of reverse causation, reinforcing the directional relationship whereby lipid alterations are likely a cause rather than a consequence of AF.

The comprehensive results, including various analysis methods, are detailed in Table **1** and Supplementary Fig. (**1**), with the IVW results serving as our primary reference. It is noteworthy that the TSMR and MR_BMA analyses concurred in their findings (Fig. **2**). Additionally, reverse MR analysis yielded negative results, suggesting no reverse causation from AF to lipidomic alterations (Fig. **3**).

### 3.2 Sensitivity Analysis to Validate Study Findings

A thorough sensitivity analysis was conducted to evaluate the robustness of the conclusion of our study. Thedetailed results of this analysis can be found in Table **1**. Within the context of AF, both the IVW and MR-Egger tests showed no evidence of heterogeneity. The MR-Egger intercept test produced a *p*-value exceeding 0.05, indicating a lack of horizontal pleiotropy and thus reinforcing the reliability of our results. Additionally, the MR-PRESSO test did not identify any pleiotropic effects, further substantiating the accuracy of our findings.

Scatter plots for the eight lipid species with significant associations are provided in Supplementary Material **1**, and leave-one-out sensitivity analyses for these species are included in Supplementary Material **2**. These analyses confirm that no individual SNP exerted a disproportionate influence on the overall MR estimates.

## 4 DISCUSSION

The intricate relationship between lipid species and AF represents a burgeoning area in cardiovascular research. Our study’s findings notably align with the “cholesterol paradox,” wherein certain lipid levels, contrary to longstanding expectations, exhibit an inverse association with AF risk [6, 29]. This paradox challenges traditional paradigms of lipid metabolism in cardiovascular diseases and highlights new avenues for therapeutic interventions.

In our analysis, specific phosphatidylcholines demonstrated differential effects on AF risk. Phosphatidylcholine (17:0_18:2) showed a protective effect, potentially due to its membrane-stabilizing properties that enhance cardiomyocyte electrical stability and reduce arrhythmic susceptibility [30]. In contrast, Phosphatidylcholine (16:0_20:5) and Phosphatidylcholine (17:0_20:4) were associated with increased risk, likely because of their diverse impacts on cellular signaling pathways and inflammatory responses [31-33]. Similarly, Phosphatidylethanolamine (18:0_20:4), which is vital for maintaining membrane curvature and fluidity, was linked to heightened AF risk, suggesting that its influence on electrical signal propagation within the heart may predispose individuals to arrhythmogenic conditions [34, 35].

Our findings extend to other lipid species as well. Elevated levels of sphingomyelin, particularly the d34:2 species, can have significant implications for cell signaling and membrane integrity, which may contribute to structural alterations in the atria and promote AF. Sphingomyelin is a crucial component of cell membranes, and its dysregulation can lead to changes in membrane fluidity and the formation of lipid rafts, which are essential for proper cell signaling and function. The disruption of these processes can result in altered cellular communication and structural remodeling of cardiac tissues [36]. One study highlights the role of ceramide, a metabolite of sphingomyelin, in the regulation of ion channels, specifically potassium channels in pancreatic β-cells. This regulation is crucial for maintaining cellular homeostasis and function. The accumulation of ceramide can alter membrane properties, potentially affecting ion channel activity and leading to cellular dysfunction [37, 38]. Similarly, in the context of cardiac cells, such disruptions could contribute to the electrical and structural remodeling associated with AF. Sterol esters, specifically Sterol ester (27:1/18:0) and Sterol ester (27:1/18:3), have been identified as risk factors for AF development [39]. These findings highlight the potential of these lipid species as biomarkers for AF risk assessment and as targets for intervention. The mechanism by which sterol esters contribute to AF risk may involve their role in inflammation and membrane fluidity, affecting the electrophysiological properties of the heart [7].

Triacylglycerol (50:4) levels were also associated with heightened AF risk. Traditionally viewed as energy storage molecules, our findings suggest that triacylglycerols may play a role in the pathogenesis of AF, potentially through mechanisms involving energy metabolism and cellular signaling [6, 40].

A major strength of our study is the integration of large-scale GWAS datasets with rigorous statistical methodologies. In particular, the application of the MR_BMA approach enabled us to simultaneously account for multiple correlated risk factors and reduce model uncertainty, thereby robustly prioritizing the most likely causal lipid determinants of AF. The consistency of our findings across multiple sensitivity analyses further reinforces the validity of our causal inferences.

Nevertheless, our study has several limitations. First, as with all MR analyses, our causal inferences depend on key assumptions-most notably, the absence of horizontal pleiotropy and minimal linkage disequilibrium among instrumental variables. Although we employed methods, such as MR-Egger and MR-PRESSO, to detect and mitigate these issues, residual pleiotropy or confounding effects cannot be entirely ruled out. Consequently, while our causal estimates remain robust across sensitivity analyses, they should be interpreted with these potential limitations in mind. Second, our analysis was based solely on GWAS data from individuals of European descent, which may limit the generalizability of our findings to populations with different genetic backgrounds, lifestyles, and environmental exposures. Third, FDR correction was not applied to adjust for multiple comparisons. It is worth noting that several published MR studies have similarly refrained from using FDR adjustments due to concerns that such corrections might be overly conservative and could mask true positive associations. We recognize that not applying FDR correction might increase the risk of type I errors, representing an additional limitation. Finally, although our bidirectional analysis did not reveal evidence of reverse causation, the considerable imbalance in sample sizes between the AF and lipidomic datasets might limit the power to detect subtle reverse effects; hence, the null results in the reverse analysis should be interpreted with this limitation in mind.

Future research should aim to broaden the diversity of study populations and investigate the temporal dynamics of lipidomic alterations in the progression of AF. Experimental studies that elucidate the precise biological mechanisms by which these lipid species influence atrial electrophysiology, inflammation, and fibrosis are also warranted. Furthermore, future work may benefit from employing negative control outcomes-traits unrelated to lipid metabolism-to further underpin the specificity of the causal relationships observed in this study. Additionally, clinical trials evaluating lipid-targeted therapies, including dietary modifications and pharmacological interventions, could pave the way for more personalized approaches to AF management.

## CONCLUSION

Our study elucidates the complex causal relationships between specific lipid species and AF, offering new insights into the etiology of AF and underscoring the potential of lipid- targeted interventions. The identification of eight lipid species significantly associated with AF riskhighlights the intricate interplay between lipid metabolism and cardiac arrhythmia, while also reinforcing the value of advanced MR methods-particularly the MR_BMA approach-in prioritizing causal determinants within highly correlated datasets. These findings not only enhance our mechanistic understanding of AF but also pave the way for future research exploring personalized lipid-targeted therapies, ultimately contributing to the advancement of cardiovascular medicine and improved patient care. In essence, our study confirms the pivotal role of lipidomics in the prevention and treatment of AF, pointing toward a promising new horizon in clinical management.

## Figures and Tables

**Fig. (1):**
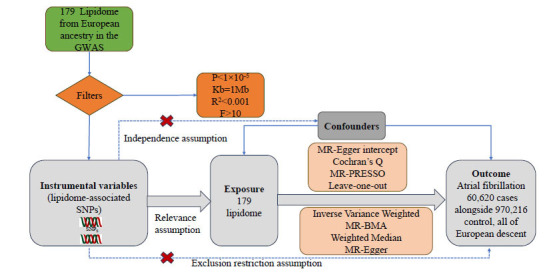
Illustrates the three primary assumptions underpinning two-sample Mendelian randomization and presents a flowchart detailing our study design, which assesses the causal relationship between lipidomic profiles and atrial fibrillation.

**Table 1:** The resluts of MR analysis between various lipidome and the risk of atrial fibrillation.

Exposure	Inverse Variance Weighted			MR-BMA			MR-Egger Intercept		MR-PRESSO Global	MR-IVW			MR-Egger		
	*P* value	OR	95% CI	*p*-value	OR	95% CI	Egger Intercept	*p*-value	*p*-value	Q	Q_df	Q_pval	Q	Q_df	Q_pval
**Glycerophospholipids**															
Phosphatidylcholine (17:0_18:2)	0.017	0.963	0.934 - 0.993	0.022	0.962	0.930-0.994	0.002	0.677	0.330	26.619	23	0.273	26.405	22	0.235
Phosphatidylcholine (16:0_20:5)	0.007	1.039	1.010 - 1.067	0.005	1.041	1.012-1.071	0.006	0.138	0.345	26.706	23	0.269	24.115	22	0.341
Phosphatidylcholine (17:0_20:4)	0.032	1.023	1.002 - 1.045	0.015	1.026	1.005-1.047	-0.001	0.739	0.445	32.186	29	0.312	32.057	28	0.272
Phosphatidylethanolamine (18:0_20:4)	0.005	1.035	1.011 - 1.060	0.005	1.035	1.011-1.061	0.001	0.892	0.612	18.212	19	0.508	18.193	18	0.443
**Sphingolipids**															
Sphingomyelin (d34:2)	0.001	1.048	1.020 - 1.077	0.036	1.035	1.002-1.069	0.004	0.289	0.531	25.234	26	0.506	24.058	25	0.516
**Glycerolipids**															
Triacylglycerol (50:4)	0.016	1.038	1.007 - 1.069	0.020	1.039	1.006-1.073	0.003	0.495	0.571	28.145	30	0.563	27.667	29	0.536
**Sterols**															
Sterol ester (27:1/18:0)	0.025	1.038	1.005 - 1.073	0.017	1.039	1.007-1.071	-0.001	0.857	0.464	24.300	24	0.445	24.265	23	0.389
Sterol ester (27:1/18:3)	0.002	1.054	1.019 - 1.090	0.001	1.059	1.023-1.097	0.002	0.772	0.627	15.743	18	0.611	15.656	17	0.548

## Data Availability

The study’s novel findings are detailed within the article and its Supplementary Materials. For additional information, please refer to the corresponding authors.

## LIST OF ABBREVIATIONS

AF: Atrial Fibrillation
MR: Mendelian Randomization
TSMR: Two-Sample Mendelian Randomization
GWAS: Genome-Wide Association Studies
SNP: Single Nucleotide Polymorphism
IVW: Inverse Variance Weighted
MR_BMA: Bayesian Model Averaging Mendelian Randomization
