# Supplementary material for: Unraveling the Lipidomic Determinants of Atrial Fibrillation: An Extensive Mendelian Randomization Study
Source: Endocr Metab Immune Disord Drug Targets. 2025 Apr 29;26:E18715303378914. doi: 10.2174/0118715303378914250418095928 (PMC13334236; doi:10.2174/0118715303378914250418095928)
Supplement: Supplementary file 1 [file EMIDDT-26-E18715303378914_SD1.pdf]

Supplementary Material

Unraveling the Lipidomic Determinants of Atrial Fibrillation: An Extensive Mendelian Randomization Study

Lingyan Ye[1,2], Dongli Lin[1,2], Feng Chen[1,2], Xiaoyong Huang[1,2] and Xianjun Wu[1,2,*]

[1]Department of Cardiology, Lishui People's Hospital, the Sixth Affiliated Hospital of Wenzhou Medical University, Lishui, Zhejiang, China; [2]Department of Cardiology, First Affiliated Hospital of Lishui University School of Medicine, Lishui, Zhejiang, China

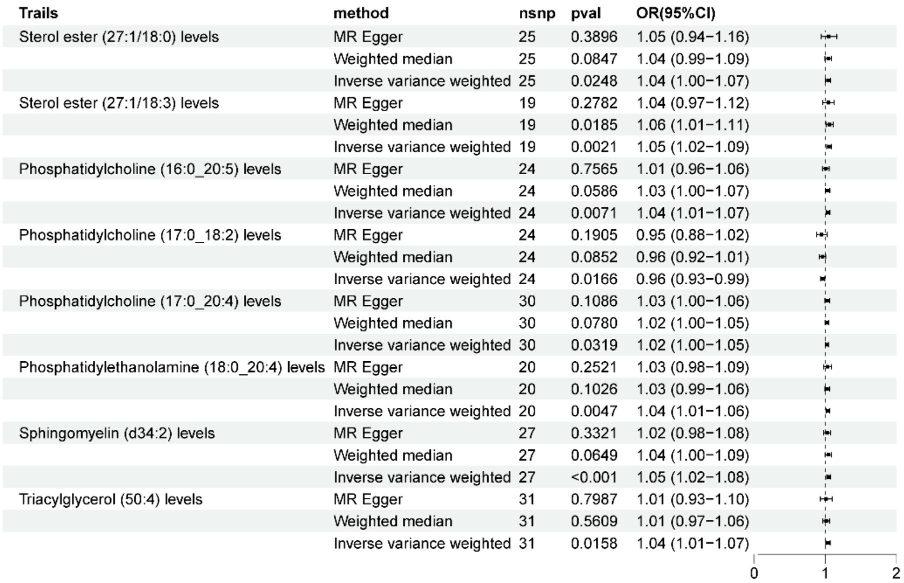

Supplement Fig. 1 A forest diagram involving three approaches to analyze the association between lipidomes and AF (Forward MR)

Supplementary 1:

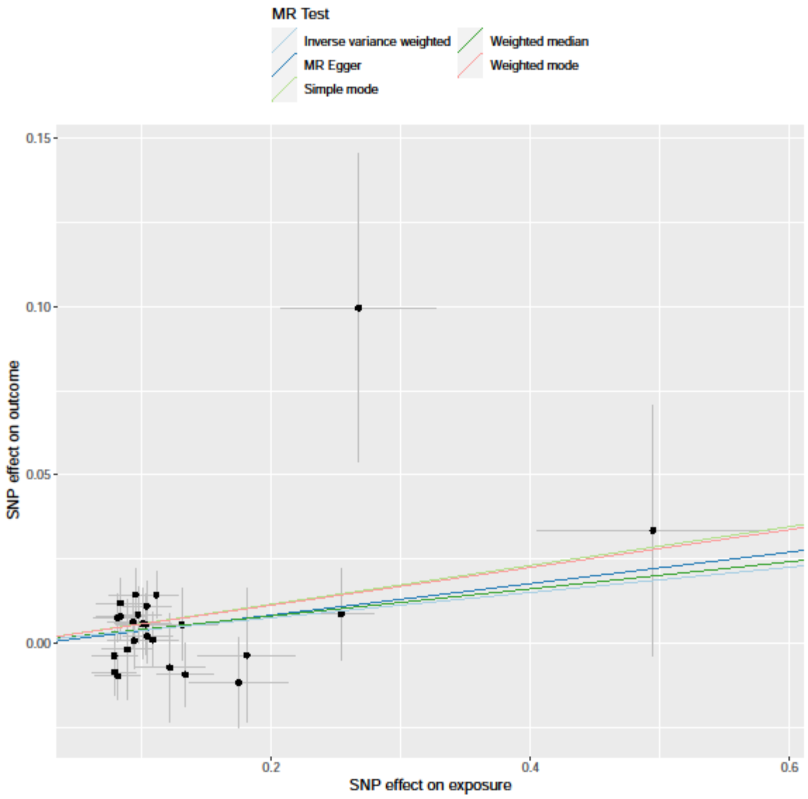

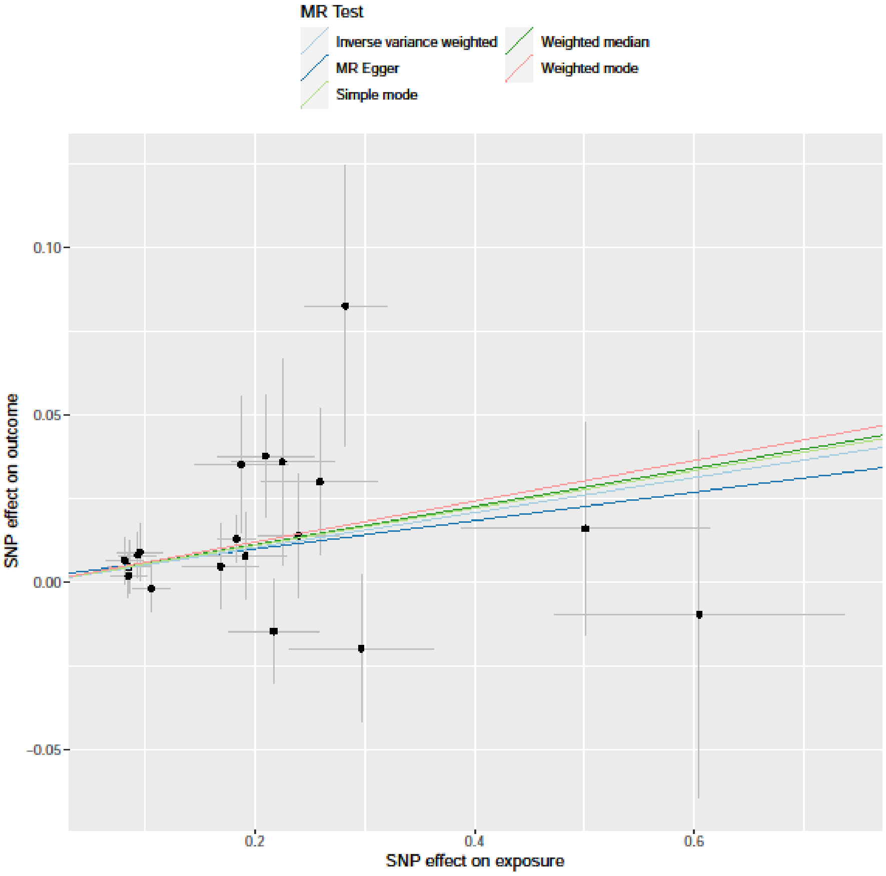

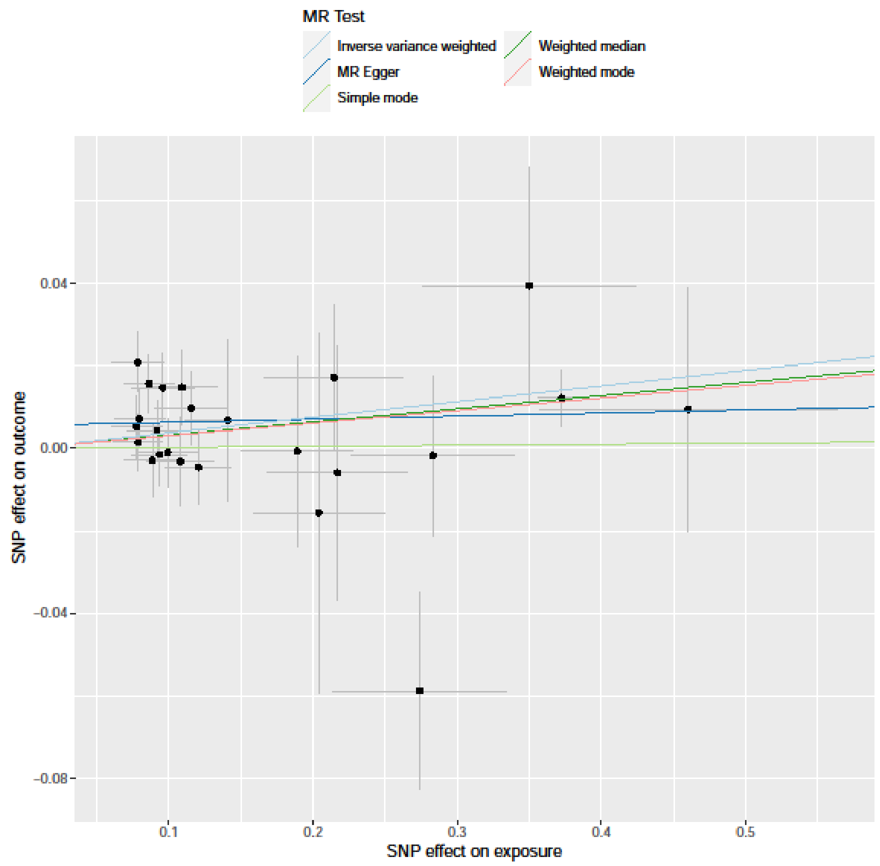
MR Test
Inverse variance weighted
MR Egger
Simple mode
Weighted median
Weighted mode
0.04
0.00
−0.04
−0.08
SNP effect on outcome
0.1
0.2
0.3
0.4
0.5
SNP effect on exposure

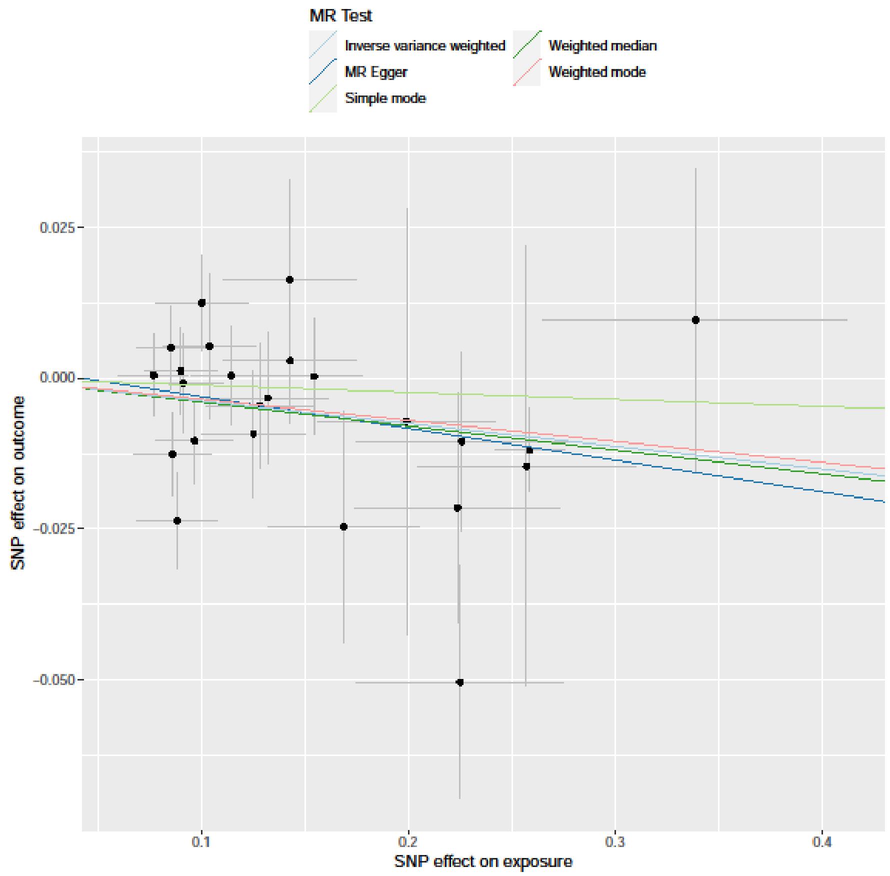
MR Test
Inverse variance weighted
MR Egger
Simple mode
Weighted median
Weighted mode
0.025
0.000
−0.025
−0.050
SNP effect on outcome
0.1
0.2
0.3
0.4
SNP effect on exposure

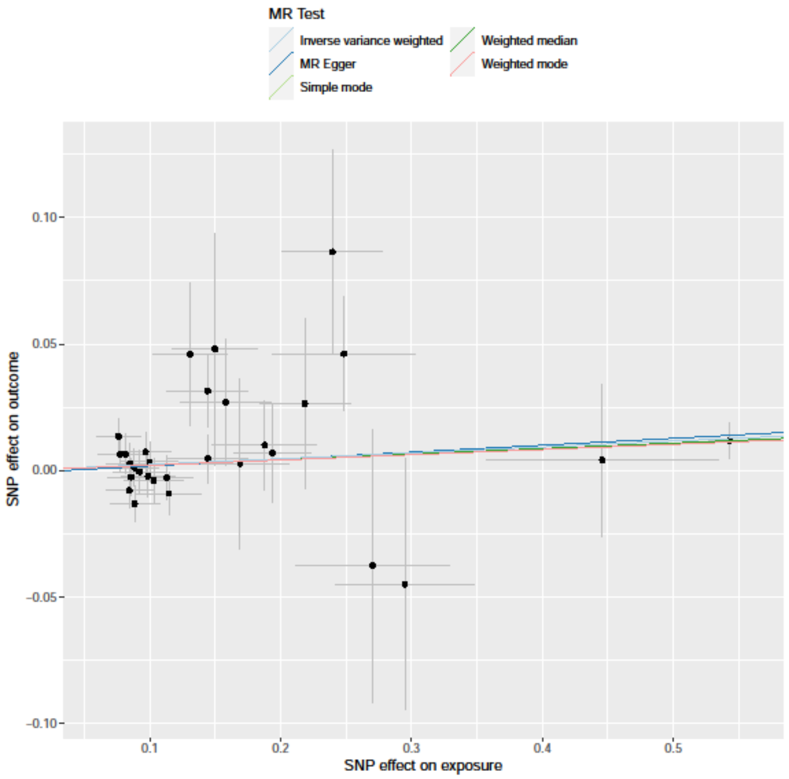
MR Test
Inverse variance weighted
MR Egger
Simple mode
Weighted median
Weighted mode
SNP effect on outcome
SNP effect on exposure
0.10
0.05
0.00
−0.05
−0.10
0.1
0.2
0.3
0.4
0.5

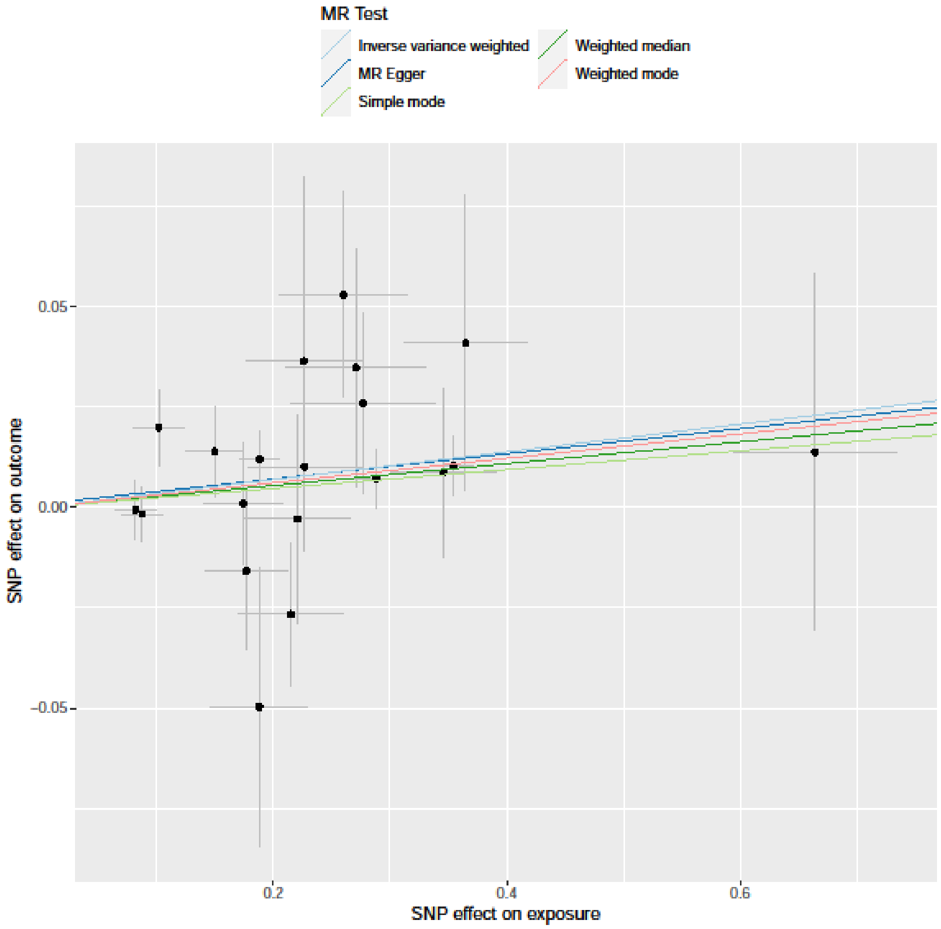
MR Test
Inverse variance weighted
MR Egger
Simple mode
Weighted median
Weighted mode
SNP effect on outcome
SNP effect on exposure
0.05
0.00
−0.05
0.2
0.4
0.6

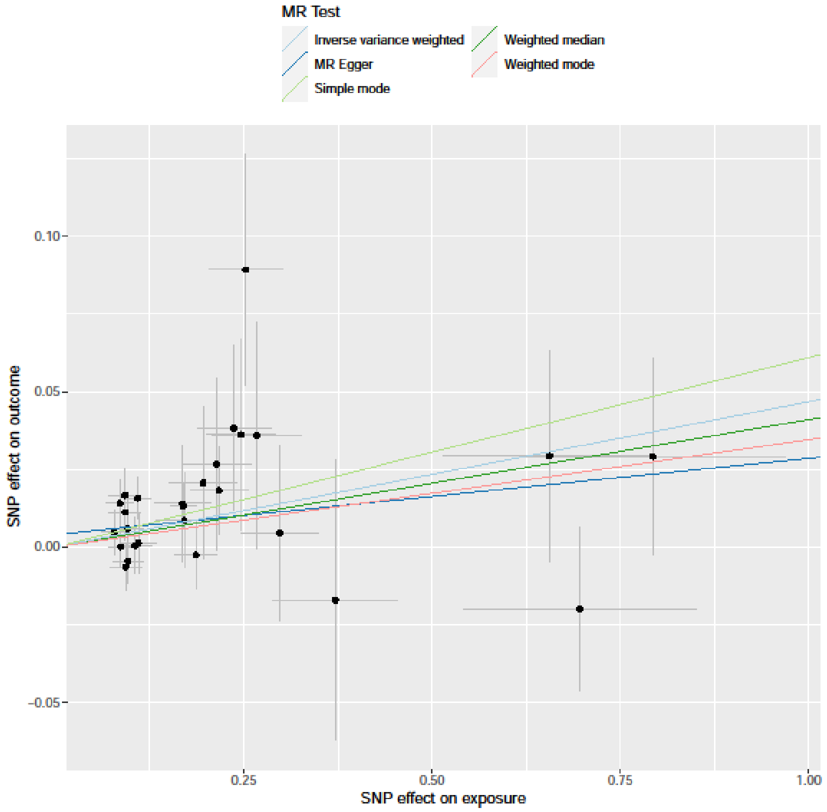
MR Test
Inverse variance weighted
MR Egger
Simple mode
Weighted median
Weighted mode
0.10
0.05
0.00
−0.05
SNP effect on outcome
0.25
0.50
0.75
1.00
SNP effect on exposure

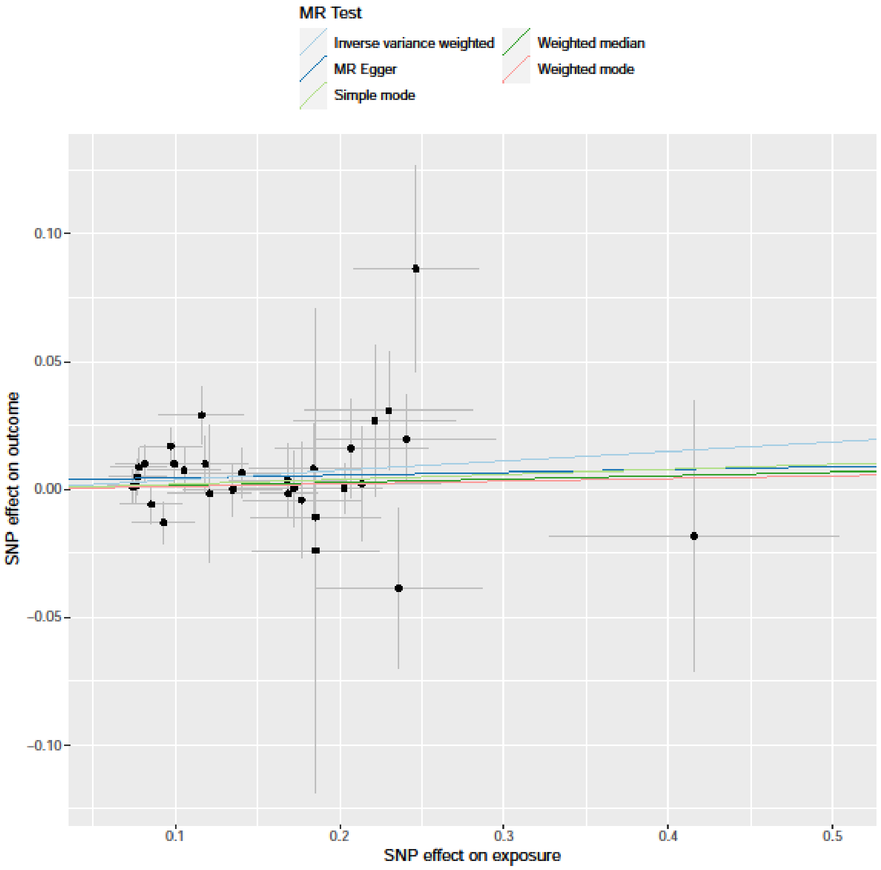
MR Test
Inverse variance weighted
MR Egger
Simple mode
Weighted median
Weighted mode
0.10
0.05
0.00
−0.05
−0.10
SNP effect on outcome
0.1
0.2
0.3
0.4
0.5
SNP effect on exposure

Supplementary 2:

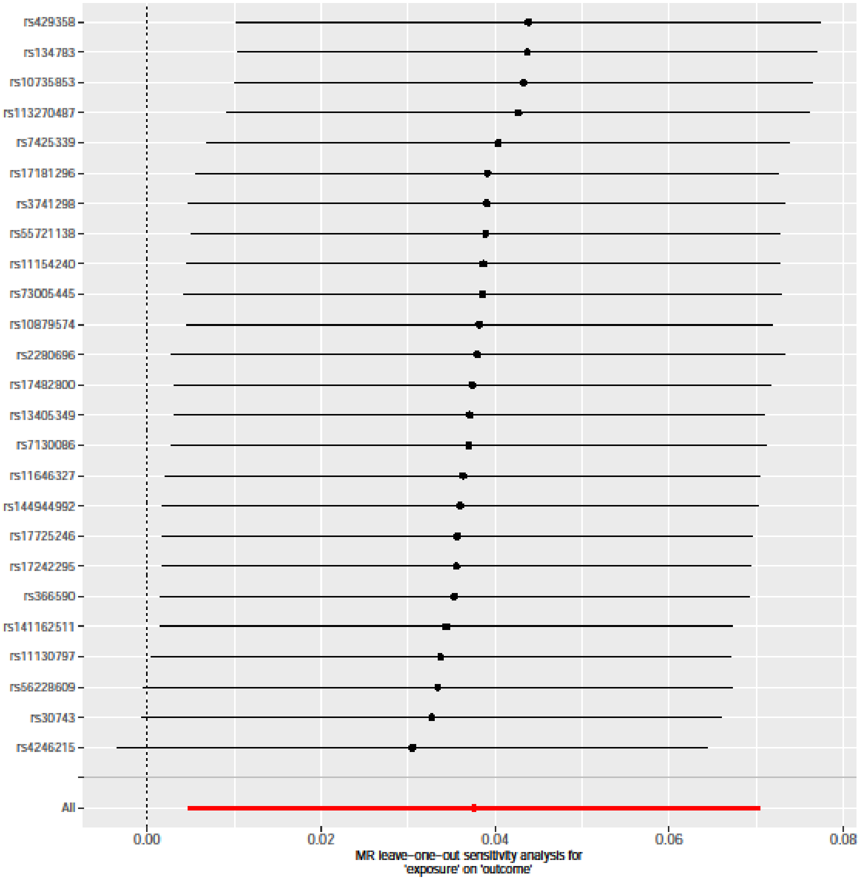

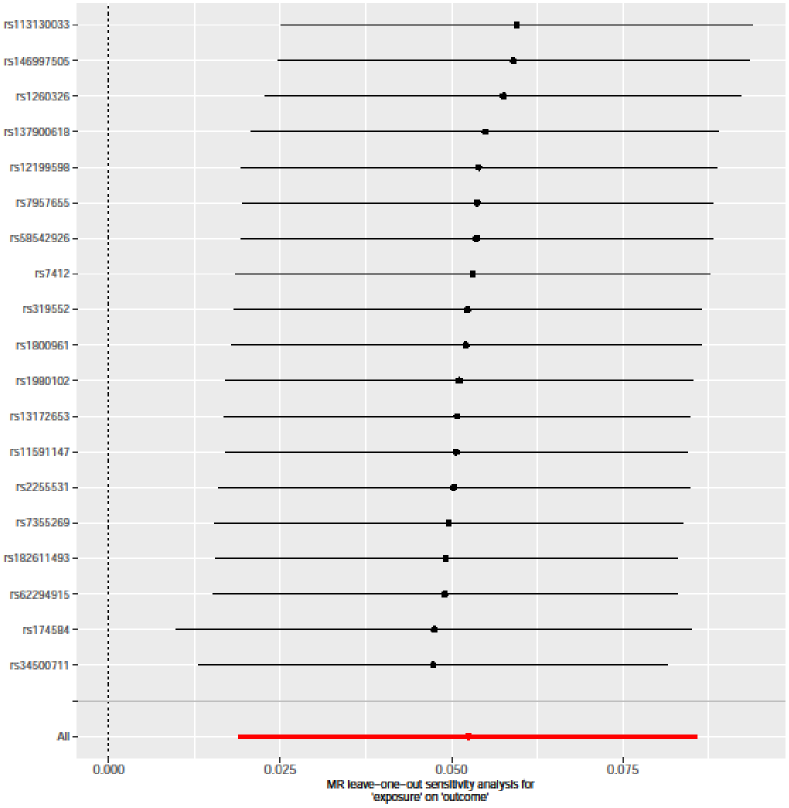

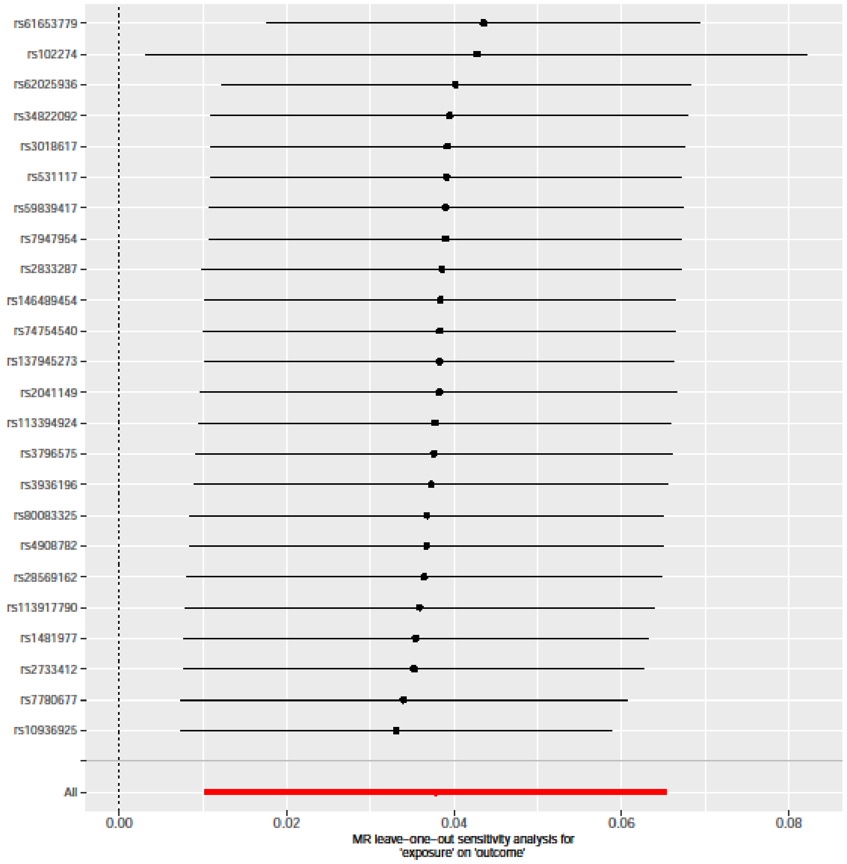
rs61653779
rs102274
rs62025936
rs34822092
rs3018617
rs531117
rs59839417
rs7947954
rs2833287
rs146489454
rs74754540
rs137945273
rs2041149
rs113394924
rs3796575
rs3936196
rs80083325
rs4908782
rs28569162
rs113917790
rs1481977
rs2733412
rs7780677
rs10936925
All
0.00
0.02
0.04
0.06
0.08
MR leave–one–out sensitivity analysis for
'exposure' on 'outcome'

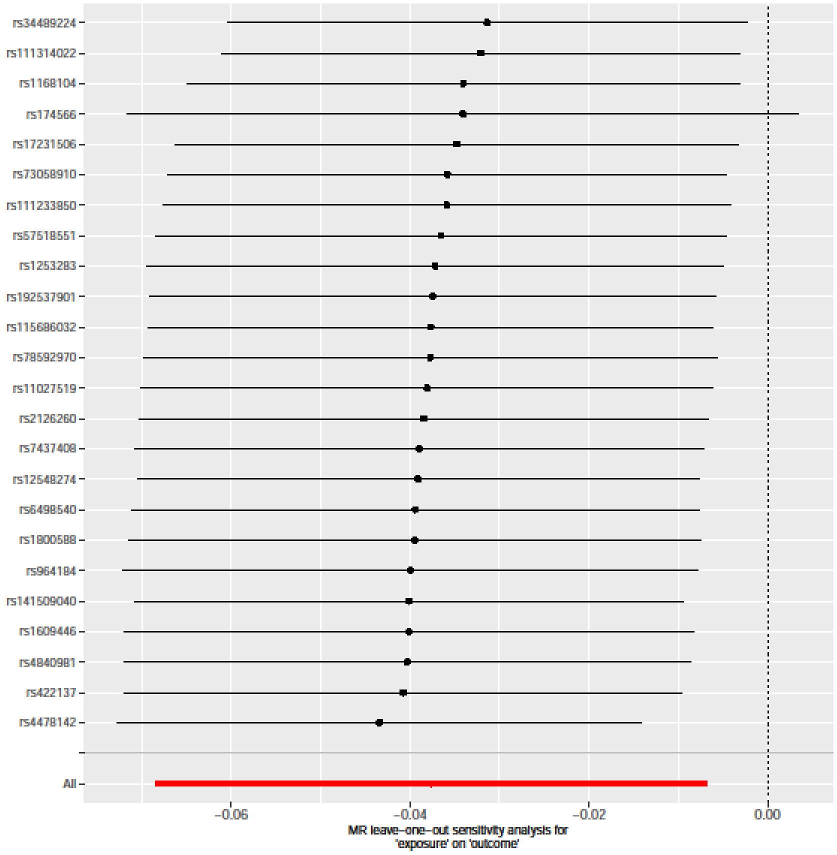
rs34489224
rs111314022
rs1168104
rs174566
rs17231506
rs73058910
rs111233850
rs57518551
rs1253283
rs192537901
rs115686032
rs78592970
rs11027519
rs2126260
rs7437408
rs12548274
rs6498540
rs1800588
rs964184
rs141509040
rs1609446
rs4840981
rs422137
rs4478142
All
−0.06
−0.04
−0.02
0.00
MR leave–one–out sensitivity analysis for
'exposure' on 'outcome'

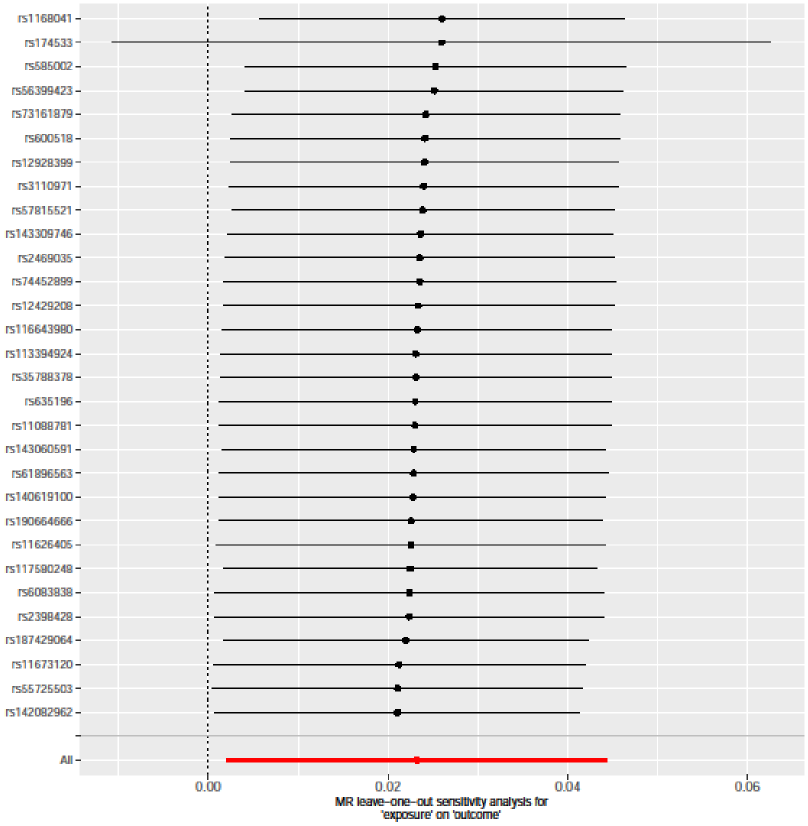
rs1168041
rs174533
rs585002
rs56399423
rs73161879
rs600518
rs12928399
rs3110971
rs57815521
rs143309746
rs2469035
rs74452899
rs12429208
rs116643980
rs113394924
rs35788378
rs635196
rs11088781
rs143060591
rs61896563
rs140619100
rs190664666
rs11626405
rs117580248
rs6083838
rs2398428
rs187429064
rs11673120
rs55725503
rs142082962
All
0.00
0.02
0.04
0.06
MR leave–one–out sensitivity analysis for
'exposure' on 'outcome'

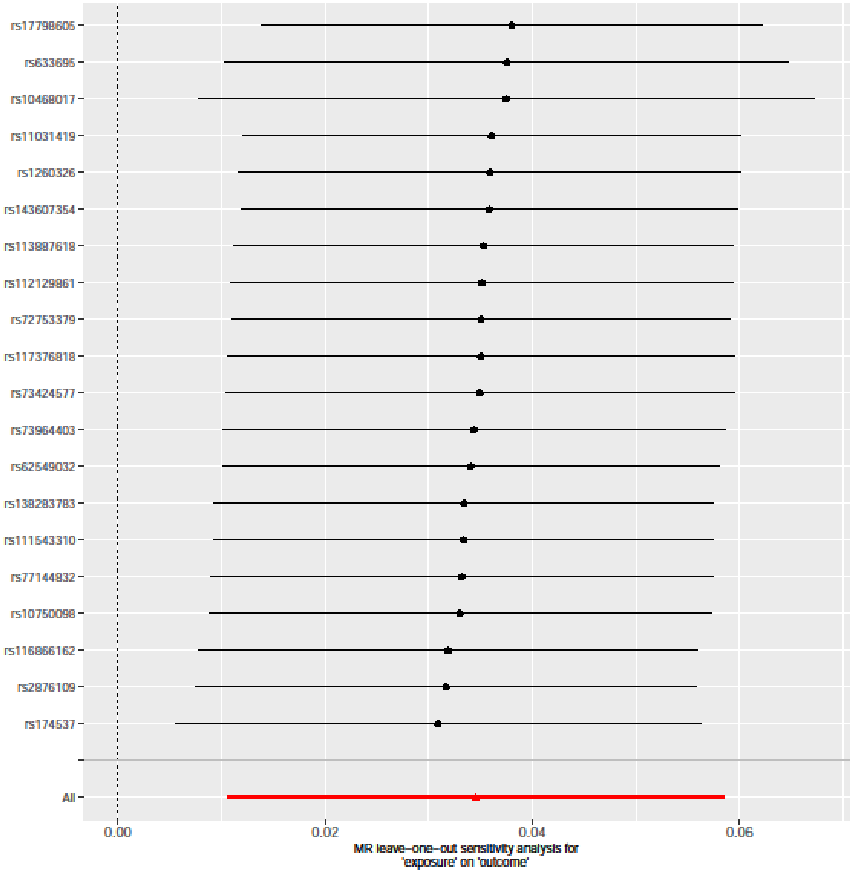
rs17798605
rs633695
rs10468017
rs11031419
rs1260326
rs143607354
rs113887618
rs112129861
rs72753379
rs117376818
rs73424577
rs73964403
rs62549032
rs138283783
rs111543310
rs77144832
rs10750098
rs116866162
rs2876109
rs174537
All
0.00
0.02
0.04
0.06
MR leave–one–out sensitivity analysis for
'exposure' on 'outcome'

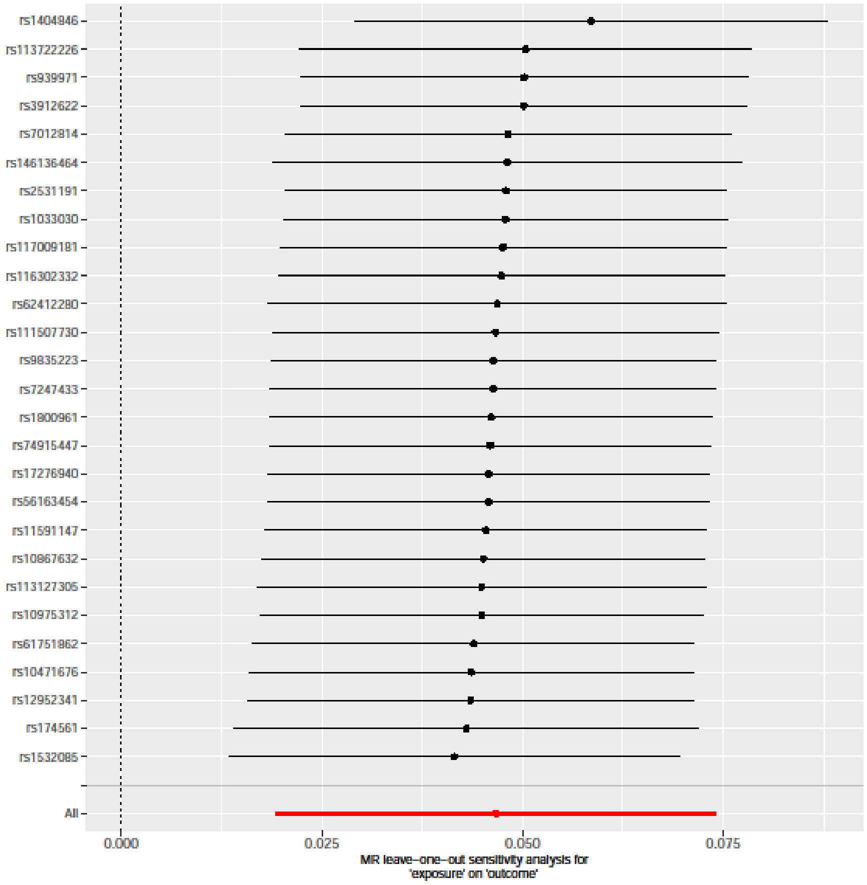
rs1404846
rs113722226
rs939971
rs3912622
rs7012814
rs146136464
rs2531191
rs1033030
rs117009181
rs116302332
rs62412280
rs111507730
rs9835223
rs7247433
rs1800961
rs74915447
rs17276940
rs56163454
rs11591147
rs10867632
rs113127305
rs10975312
rs61751862
rs10471676
rs12952341
rs174561
rs1532085
All
0.000
0.025
0.050
0.075
MR leave–one–out sensitivity analysis for
'exposure' on 'outcome'

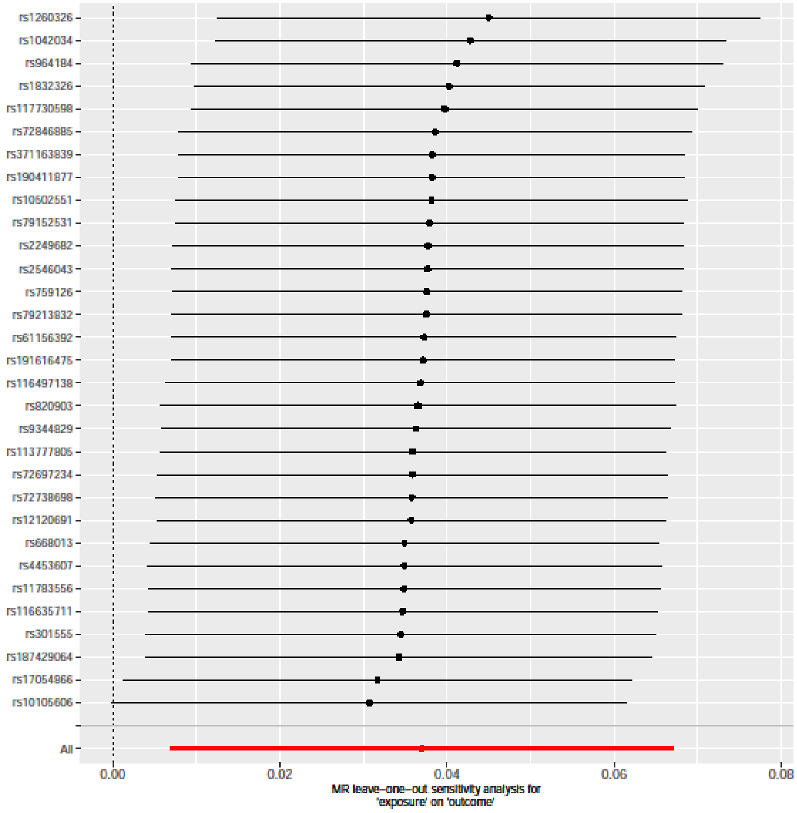
rs1260326
rs1042034
rs964184
rs1832326
rs117730598
rs72846885
rs371163839
rs190411877
rs10502551
rs79152531
rs2249682
rs2546043
rs759126
rs79213832
rs61156392
rs191616475
rs116497138
rs820903
rs9344829
rs113777806
rs72697234
rs72738698
rs12120691
rs668013
rs4453607
rs11783556
rs116635711
rs301555
rs187429064
rs17054866
rs10105606
All
0.00
0.02
0.04
0.06
0.08
MR leave–one–out sensitivity analysis for
'exposure' on 'outcome'

Supplement Table 1. Lipidome Index Number.

Id	ReportedTrait	EfoTraits
GCST90277238	Sterol ester (27:1/14:0) levels	level of Sterol ester (27:1/14:0) in blood serum
GCST90277239	Sterol ester (27:1/15:0) levels	level of Sterol ester (27:1/15:0) in blood serum
GCST90277240	Sterol ester (27:1/16:0) levels	level of Sterol ester (27:1/16:0) in blood serum
GCST90277241	Sterol ester (27:1/16:1) levels	level of Sterol ester (27:1/16:1) in blood serum
GCST90277242	Sterol ester (27:1/17:0) levels	level of Sterol ester (27:1/17:0) in blood serum
GCST90277243	Sterol ester (27:1/17:1) levels	esterified cholesterol measurement
GCST90277244	Sterol ester (27:1/18:0) levels	level of Sterol ester (27:1/18:0) in blood serum
GCST90277245	Sterol ester (27:1/18:1) levels	level of Sterol ester (27:1/18:1) in blood serum
GCST90277246	Sterol ester (27:1/18:2) levels	level of Sterol ester (27:1/18:2) in blood serum
GCST90277247	Sterol ester (27:1/18:3) levels	level of Sterol ester (27:1/18:3) in blood serum
GCST90277248	Sterol ester (27:1/20:2) levels	level of Sterol ester (27:1/20:2) in blood serum
GCST90277249	Sterol ester (27:1/20:3) levels	level of Sterol ester (27:1/20:3) in blood serum
GCST90277250	Sterol ester (27:1/20:4) levels	level of Sterol ester (27:1/20:4) in blood serum
GCST90277251	Sterol ester (27:1/20:5) levels	level of Sterol ester (27:1/20:5) in blood serum
GCST90277252	Sterol ester (27:1/22:6) levels	level of Sterol ester (27:1/22:6) in blood serum
GCST90277253	Ceramide (d40:1) levels	level of Ceramide (d40:1) in blood serum
GCST90277254	Ceramide (d40:2) levels	level of Ceramide (d40:2) in blood serum
GCST90277255	Ceramide (d42:1) levels	level of Ceramide (d42:1) in blood serum
GCST90277256	Ceramide (d42:2) levels	level of Ceramide (d42:2) in blood serum
GCST90277257	Cholesterol levels	total cholesterol measurement
GCST90277258	Diacylglycerol (16:0_18:1) levels	level of Diacylglycerol (16:0_18:1) in blood serum
GCST90277259	Diacylglycerol (16:0_18:2) levels	level of Diacylglycerol (16:0_18:2) in blood serum

GCST90277260	Diacylglycerol (16:1_18:1) levels	level of Diacylglycerol (16:1_18:1) in blood serum
GCST90277261	Diacylglycerol (18:1_18:1) levels	level of Diacylglycerol (18:1_18:1) in blood serum
GCST90277262	Diacylglycerol (18:1_18:2) levels	level of Diacylglycerol (18:1_18:2) in blood serum
GCST90277338	Phosphatidylcholine (O-18:1_18:2) levels	level of Phosphatidylcholine (O-18:1_18:2) in blood serum
GCST90277339	Phosphatidylcholine (O-18:1_20:3) levels	level of Phosphatidylcholine (O-18:1_20:3) in blood serum
GCST90277340	Phosphatidylcholine (O-18:1_20:4) levels	level of Phosphatidylcholine (O-18:1_20:4) in blood serum
GCST90277341	Phosphatidylcholine (O-18:2_16:0) levels	level of Phosphatidylcholine (O-18:2_16:0) in blood serum
GCST90277342	Phosphatidylcholine (O-18:2_18:1) levels	level of Phosphatidylcholine (O-18:2_18:1) in blood serum
GCST90277343	Phosphatidylcholine (O-18:2_18:2) levels	level of Phosphatidylcholine (O-18:2_18:2) in blood serum
GCST90277344	Phosphatidylcholine (O-18:2_20:4) levels	level of Phosphatidylcholine (O-18:2_20:4) in blood serum
GCST90277345	Phosphatidylethanolamine (16:0_18:2) levels	level of Phosphatidylethanolamine (16:0_18:2) in blood serum
GCST90277346	Phosphatidylethanolamine (16:0_20:4) levels	level of Phosphatidylethanolamine (16:0_20:4) in blood serum
GCST90277347	Phosphatidylethanolamine (18:0_18:2) levels	level of Phosphatidylethanolamine (18:0_18:2) in blood serum
GCST90277348	Phosphatidylethanolamine (18:0_20:4) levels	level of Phosphatidylethanolamine (18:0_20:4) in blood serum
GCST90277349	Phosphatidylethanolamine (18:1_18:1) levels	level of Phosphatidylethanolamine (18:1_18:1) in blood serum
GCST90277350	Phosphatidylethanolamine (O-16:1_18:2) levels	level of Phosphatidylethanolamine (O-16:1_18:2) in blood serum
GCST90277351	Phosphatidylethanolamine (O-16:1_20:4) levels	level of Phosphatidylethanolamine (O-16:1_20:4) in blood serum
GCST90277352	Phosphatidylethanolamine (O-16:1_22:5) levels	level of Phosphatidylethanolamine (O-16:1_22:5) in blood serum
GCST90277353	Phosphatidylethanolamine (O-18:1_18:2) levels	level of Phosphatidylethanolamine (O-18:1_18:2) in blood serum
GCST90277354	Phosphatidylethanolamine (O-18:1_20:4) levels	level of Phosphatidylethanolamine (O-18:1_20:4) in blood serum
GCST90277355	Phosphatidylethanolamine (O-18:2_18:1) levels	level of Phosphatidylethanolamine (O-18:2_18:1) in blood serum
GCST90277356	Phosphatidylethanolamine (O-18:2_18:2) levels	level of Phosphatidylethanolamine (O-18:2_18:2) in blood serum

GCST90277357	Phosphatidylethanolamine (O-18:2_20:4) levels	level of Phosphatidylethanolamine (O-18:2_20:4) in blood serum
GCST90277358	Phosphatidylinositol (16:0_18:1) levels	level of Phosphatidylinositol (16:0_18:1) in blood serum
GCST90277359	Phosphatidylinositol (16:0_18:2) levels	level of Phosphatidylinositol (16:0_18:2) in blood serum
GCST90277360	Phosphatidylinositol (16:0_20:4) levels	level of Phosphatidylinositol (16:0_20:4) in blood serum
GCST90277361	Phosphatidylinositol (18:0_18:1) levels	level of Phosphatidylinositol (18:0_18:1) in blood serum
GCST90277362	Phosphatidylinositol (18:0_18:2) levels	level of Phosphatidylinositol (18:0_18:2) in blood serum
GCST90277288	Phosphatidylcholine (16:0_20:5) levels	level of Phosphatidylcholine (16:0_20:5) in blood serum
GCST90277289	Phosphatidylcholine (16:0_22:4) levels	level of Phosphatidylcholine (16:0_22:4) in blood serum
GCST90277290	Phosphatidylcholine (16:0_22:5) levels	level of Phosphatidylcholine (16:0_22:5) in blood serum
GCST90277291	Phosphatidylcholine (16:0_22:6) levels	level of Phosphatidylcholine (16:0_22:6) in blood serum
GCST90277292	Phosphatidylcholine (16:1_18:0) levels	level of Phosphatidylcholine (16:1_18:0) in blood serum
GCST90277293	Phosphatidylcholine (16:1_18:1) levels	level of Phosphatidylcholine (16:1_18:1) in blood serum
GCST90277294	Phosphatidylcholine (16:1_18:2) levels	level of Phosphatidylcholine (16:1_18:2) in blood serum
GCST90277295	Phosphatidylcholine (16:1_20:4) levels	level of Phosphatidylcholine (16:1_20:4) in blood serum
GCST90277296	Phosphatidylcholine (17:0_18:1) levels	level of Phosphatidylcholine (17:0_18:1) in blood serum
GCST90277297	Phosphatidylcholine (17:0_18:2) levels	level of Phosphatidylcholine (17:0_18:2) in blood serum
GCST90277298	Phosphatidylcholine (17:0_20:4) levels	level of Phosphatidylcholine (17:0_20:4) in blood serum
GCST90277299	Phosphatidylcholine (18:0_18:1) levels	level of Phosphatidylcholine (18:0_18:1) in blood serum
GCST90277300	Phosphatidylcholine (18:0_18:2) levels	level of Phosphatidylcholine (18:0_18:2) in blood serum
GCST90277301	Phosphatidylcholine (18:0_18:3) levels	level of Phosphatidylcholine (18:0_18:3) in blood serum
GCST90277302	Phosphatidylcholine (18:0_20:2) levels	level of Phosphatidylcholine (18:0_20:2) in blood serum
GCST90277303	Phosphatidylcholine (18:0_20:3) levels	level of Phosphatidylcholine (18:0_20:3) in blood serum

GCST90277304	Phosphatidylcholine (18:0_20:4) levels	level of Phosphatidylcholine (18:0_20:4) in blood serum
GCST90277305	Phosphatidylcholine (18:0_20:5) levels	level of Phosphatidylcholine (18:0_20:5) in blood serum
GCST90277306	Phosphatidylcholine (18:0_22:5) levels	level of Phosphatidylcholine (18:0_22:5) in blood serum
GCST90277307	Phosphatidylcholine (18:0_22:6) levels	level of Phosphatidylcholine (18:0_22:6) in blood serum
GCST90277308	Phosphatidylcholine (18:1_18:1) levels	level of Phosphatidylcholine (18:1_18:1) in blood serum
GCST90277309	Phosphatidylcholine (18:1_18:2) levels	level of Phosphatidylcholine (18:1_18:2) in blood serum
GCST90277310	Phosphatidylcholine (18:1_18:3) levels	level of Phosphatidylcholine (18:1_18:3) in blood serum
GCST90277311	Phosphatidylcholine (18:1_20:2) levels	level of Phosphatidylcholine (18:1_20:2) in blood serum
GCST90277312	Phosphatidylcholine (18:1_20:3) levels	level of Phosphatidylcholine (18:1_20:3) in blood serum
GCST90277313	Phosphatidylcholine (18:1_20:4) levels	level of Phosphatidylcholine (18:1_20:4) in blood serum
GCST90277314	Phosphatidylcholine (18:2_18:2) levels	level of Phosphatidylcholine (18:2_18:2) in blood serum
GCST90277315	Phosphatidylcholine (18:2_20:1) levels	level of Phosphatidylcholine (18:2_20:1) in blood serum
GCST90277316	Phosphatidylcholine (18:2_20:3) levels	level of Phosphatidylcholine (18:2_20:3) in blood serum
GCST90277317	Phosphatidylcholine (18:2_20:4) levels	level of Phosphatidylcholine (18:2_20:4) in blood serum
GCST90277318	Phosphatidylcholine (O-16:0_16:0) levels	level of Phosphatidylcholine (O-16:0_16:0) in blood serum
GCST90277319	Phosphatidylcholine (O-16:0_16:1) levels	level of Phosphatidylcholine (O-16:0_16:1) in blood serum
GCST90277320	Phosphatidylcholine (O-16:0_18:1) levels	level of Phosphatidylcholine (O-16:0_18:1) in blood serum
GCST90277321	Phosphatidylcholine (O-16:0_18:2) levels	level of Phosphatidylcholine (O-16:0_18:2) in blood serum
GCST90277322	Phosphatidylcholine (O-16:0_20:3) levels	level of Phosphatidylcholine (O-16:0_20:3) in blood serum
GCST90277323	Phosphatidylcholine (O-16:0_20:4) levels	level of Phosphatidylcholine (O-16:0_20:4) in blood serum
GCST90277324	Phosphatidylcholine (O-16:0_22:5) levels	level of Phosphatidylcholine (O-16:0_22:5) in blood serum
GCST90277325	Phosphatidylcholine (O-16:1_16:0) levels	level of Phosphatidylcholine (O-16:1_16:0) in blood serum

GCST90277326	Phosphatidylcholine (O-16:1_18:0) levels	level of Phosphatidylcholine (O-16:1_18:0) in blood serum
GCST90277327	Phosphatidylcholine (O-16:1_18:1) levels	level of Phosphatidylcholine (O-16:1_18:1) in blood serum
GCST90277328	Phosphatidylcholine (O-16:1_18:2) levels	level of Phosphatidylcholine (O-16:1_18:2) in blood serum
GCST90277329	Phosphatidylcholine (O-16:1_20:3) levels	level of Phosphatidylcholine (O-16:1_20:3) in blood serum
GCST90277330	Phosphatidylcholine (O-16:1_20:4) levels	level of Phosphatidylcholine (O-16:1_20:4) in blood serum
GCST90277331	Phosphatidylcholine (O-16:2_18:0) levels	level of phosphatidylcholine
GCST90277332	Phosphatidylcholine (O-17:0_15:0) levels	level of Phosphatidylcholine (O-17:0_15:0) in blood serum
GCST90277333	Phosphatidylcholine (O-17:0_17:1) levels	level of phosphatidylcholine
GCST90277334	Phosphatidylcholine (O-18:0_14:0) levels	level of Phosphatidylcholine (O-18:0_14:0) in blood serum
GCST90277335	Phosphatidylcholine (O-18:0_16:1) levels	level of Phosphatidylcholine (O-18:0_16:1) in blood serum
GCST90277336	Phosphatidylcholine (O-18:0_20:4) levels	level of Phosphatidylcholine (O-18:0_20:4) in blood serum
GCST90277337	Phosphatidylcholine (O-18:1_16:0) levels	level of Phosphatidylcholine (O-18:1_16:0) in blood serum
GCST90277363	Phosphatidylinositol (18:0_20:3) levels	level of Phosphatidylinositol (18:0_20:3) in blood serum
GCST90277364	Phosphatidylinositol (18:0_20:4) levels	level of Phosphatidylinositol (18:0_20:4) in blood serum
GCST90277365	Phosphatidylinositol (18:1_18:1) levels	level of Phosphatidylinositol (18:1_18:1) in blood serum
GCST90277366	Phosphatidylinositol (18:1_18:2) levels	level of Phosphatidylinositol (18:1_18:2) in blood serum
GCST90277367	Phosphatidylinositol (18:1_20:4) levels	level of Phosphatidylinositol (18:1_20:4) in blood serum
GCST90277368	Sphingomyelin (d32:1) levels	level of Sphingomyelin (d32:1) in blood serum
GCST90277369	Sphingomyelin (d34:0) levels	level of Sphingomyelin (d34:0) in blood serum
GCST90277370	Sphingomyelin (d34:1) levels	level of Sphingomyelin (d34:1) in blood serum
GCST90277371	Sphingomyelin (d34:2) levels	level of Sphingomyelin (d34:2) in blood serum
GCST90277372	Sphingomyelin (d36:1) levels	level of Sphingomyelin (d36:1) in blood serum
GCST90277373	Sphingomyelin (d36:2) levels	level of Sphingomyelin (d36:2) in blood serum

GCST90277374	Sphingomyelin (d38:1) levels	level of Sphingomyelin (d38:1) in blood serum
GCST90277375	Sphingomyelin (d38:2) levels	level of Sphingomyelin (d38:2) in blood serum
GCST90277376	Sphingomyelin (d40:1) levels	level of Sphingomyelin (d40:1) in blood serum
GCST90277377	Sphingomyelin (d40:2) levels	level of Sphingomyelin (d40:2) in blood serum
GCST90277378	Sphingomyelin (d42:2) levels	level of Sphingomyelin (d42:2) in blood serum
GCST90277379	Triacylglycerol (46:1) levels	triacylglycerol 46:1 measurement
GCST90277380	Triacylglycerol (46:2) levels	triacylglycerol 46:2 measurement
GCST90277381	Triacylglycerol (48:0) levels	triacylglycerol 48:0 measurement
GCST90277382	Triacylglycerol (48:1) levels	triacylglycerol 48:1 measurement
GCST90277383	Triacylglycerol (48:2) levels	triacylglycerol 48:2 measurement
GCST90277384	Triacylglycerol (48:3) levels	triacylglycerol 48:3 measurement
GCST90277385	Triacylglycerol (49:1) levels	triacylglycerol 49:1 measurement
GCST90277386	Triacylglycerol (49:2) levels	level of Triacylglycerol (49:2) in blood serum
GCST90277387	Triacylglycerol (50:1) levels	triacylglycerol 50:1 measurement
GCST90277388	Triacylglycerol (50:2) levels	triacylglycerol 50:2 measurement
GCST90277389	Triacylglycerol (50:3) levels	triacylglycerol 50:3 measurement
GCST90277390	Triacylglycerol (50:4) levels	triacylglycerol 50:4 measurement
GCST90277391	Triacylglycerol (50:5) levels	triacylglycerol 50:5 measurement
GCST90277392	Triacylglycerol (51:1) levels	triacylglycerol 51:1 measurement
GCST90277393	Triacylglycerol (51:2) levels	triacylglycerol 51:2 measurement
GCST90277394	Triacylglycerol (51:3) levels	level of Triacylglycerol (51:3) in blood serum
GCST90277395	Triacylglycerol (51:4) levels	level of Triacylglycerol (51:4) in blood serum
GCST90277396	Triacylglycerol (52:2) levels	triacylglycerol 52:2 measurement
GCST90277397	Triacylglycerol (52:3) levels	triacylglycerol 52:3 measurement
GCST90277398	Triacylglycerol (52:4) levels	triacylglycerol 52:4 measurement
GCST90277399	Triacylglycerol (52:5) levels	triacylglycerol 52:5 measurement
GCST90277400	Triacylglycerol (52:6) levels	triacylglycerol 52:6 measurement
GCST90277401	Triacylglycerol (53:2) levels	triacylglycerol 53:2 measurement
GCST90277402	Triacylglycerol (53:3) levels	level of Triacylglycerol (53:3) in blood serum
GCST90277403	Triacylglycerol (53:4) levels	level of Triacylglycerol (53:4) in blood serum
GCST90277404	Triacylglycerol (54:3) levels	triacylglycerol 54:3 measurement
GCST90277405	Triacylglycerol (54:4) levels	triacylglycerol 54:4 measurement
GCST90277406	Triacylglycerol (54:5) levels	triacylglycerol 54:5 measurement
GCST90277407	Triacylglycerol (54:6) levels	triacylglycerol 54:6 measurement

GCST90277408	Triacylglycerol (54:7) levels	triacylglycerol 54:7 measurement
GCST90277409	Triacylglycerol (56:3) levels	triacylglycerol 56:3 measurement
GCST90277410	Triacylglycerol (56:4) levels	triacylglycerol 56:4 measurement
GCST90277411	Triacylglycerol (56:5) levels	triacylglycerol 56:5 measurement
GCST90277412	Triacylglycerol (56:6) levels	triacylglycerol 56:6 measurement
GCST90277413	Triacylglycerol (56:7) levels	triacylglycerol 56:7 measurement
GCST90277414	Triacylglycerol (56:8) levels	triacylglycerol 56:8 measurement
GCST90277415	Triacylglycerol (58:7) levels	triacylglycerol 58:7 measurement
GCST90277416	Triacylglycerol (58:8) levels	triacylglycerol 58:8 measurement
GCST90277263	Diacylglycerol (18:1_18:3) levels	level of Diacylglycerol (18:1_18:3) in blood serum
GCST90277264	Phosphatidylcholine (16:0_0:0) levels	level of Phosphatidylcholine (16:0_0:0) in blood serum
GCST90277265	Phosphatidylcholine (18:0_0:0) levels	level of Phosphatidylcholine (18:0_0:0) in blood serum
GCST90277266	Phosphatidylcholine (18:1_0:0) levels	level of Phosphatidylcholine (18:1_0:0) in blood serum
GCST90277267	Phosphatidylcholine (18:2_0:0) levels	level of Phosphatidylcholine (18:2_0:0) in blood serum
GCST90277268	Phosphatidylcholine (20:4_0:0) levels	level of Phosphatidylcholine (20:4_0:0) in blood serum
GCST90277269	Phosphatidylethanolamine (18:0_0:0) levels	level of Phosphatidylethanolamine (18:0_0:0) in blood serum
GCST90277270	Phosphatidylethanolamine (18:1_0:0) levels	level of Phosphatidylethanolamine (18:1_0:0) in blood serum
GCST90277271	Phosphatidylethanolamine (18:2_0:0) levels	level of Phosphatidylethanolamine (18:2_0:0) in blood serum
GCST90277272	Phosphatidylcholine (14:0_16:0) levels	level of Phosphatidylcholine (14:0_16:0) in blood serum
GCST90277273	Phosphatidylcholine (14:0_18:1) levels	level of Phosphatidylcholine (14:0_18:1) in blood serum
GCST90277274	Phosphatidylcholine (14:0_18:2) levels	level of Phosphatidylcholine (14:0_18:2) in blood serum
GCST90277275	Phosphatidylcholine (15:0_18:1) levels	level of Phosphatidylcholine (15:0_18:1) in blood serum
GCST90277276	Phosphatidylcholine (15:0_18:2) levels	level of Phosphatidylcholine (15:0_18:2) in blood serum
GCST90277277	Phosphatidylcholine (16:0_16:0) levels	level of Phosphatidylcholine (16:0_16:0) in blood serum
GCST90277278	Phosphatidylcholine (16:0_16:1) levels	level of Phosphatidylcholine (16:0_16:1) in blood serum
GCST90277279	Phosphatidylcholine (16:0_17:1) levels	level of phosphatidylcholine
GCST90277280	Phosphatidylcholine (16:0_18:0) levels	level of Phosphatidylcholine (16:0_18:0) in blood serum

GCST90277281	Phosphatidylcholine (16:0_18:1) levels	level of Phosphatidylcholine (16:0_18:1) in blood serum
GCST90277282	Phosphatidylcholine (16:0_18:2) levels	level of Phosphatidylcholine (16:0_18:2) in blood serum
GCST90277283	Phosphatidylcholine (16:0_18:3) levels	level of Phosphatidylcholine (16:0_18:3) in blood serum
GCST90277284	Phosphatidylcholine (16:0_20:1) levels	level of Phosphatidylcholine (16:0_20:1) in blood serum
GCST90277285	Phosphatidylcholine (16:0_20:2) levels	level of Phosphatidylcholine (16:0_20:2) in blood serum
GCST90277286	Phosphatidylcholine (16:0_20:3) levels	level of Phosphatidylcholine (16:0_20:3) in blood serum
GCST90277287	Phosphatidylcholine (16:0_20:4) levels	level of Phosphatidylcholine (16:0_20:4) in blood serum
